# Evaluation of Primary Allied Health Care in Patients Recovering From COVID-19 at 6-Month Follow-up: Dutch Nationwide Prospective Cohort Study

**DOI:** 10.2196/44155

**Published:** 2023-10-20

**Authors:** Anne I Slotegraaf, Marissa H G Gerards, Arie C Verburg, Marian A E de van der Schueren, Hinke M Kruizenga, Maud J L Graff, Edith H C Cup, Johanna G Kalf, Antoine F Lenssen, Willemijn M Meijer, Renée A Kool, Rob A de Bie, Philip J van der Wees, Thomas J Hoogeboom, Dutch Consortium Allied Healthcare COVID-19

**Affiliations:** 1 Division of Human Nutrition and Health Wageningen University and Research Wageningen Netherlands; 2 Department of Epidemiology, Care and Public Health Institute Faculty of Health, Medicine and Life Sciences Maastricht University Maastricht Netherlands; 3 Department of Physical Therapy Maastricht University Medical Centre Maastricht Netherlands; 4 IQ Healthcare Radboud Institute for Health Sciences Radboud University Medical Centre Nijmegen Netherlands; 5 Department of Nutrition, Dietetics and Lifestyle HAN University of Applied Sciences Nijmegen Netherlands; 6 Department of Nutrition and Dietetics Amsterdam University Medical Centers Amsterdam Netherlands; 7 Department of Rehabilitation Donders Institute for Brain, Cognition and Behaviour Radboud University Medical Centre Nijmegen Netherlands; 8 Netherlands Institute for Health Services Research Nivel Utrecht Netherlands; 9 Lung Foundation Netherlands Amersfoort Netherlands; 10 See Acknowledgments

**Keywords:** COVID-19, allied health care, primary care, care, patient, physical, nutritional, cognitive, mental functioning, support, recovery, diet, exercise, exercise therapist, physical therapist, speech therapist, language, descriptive statistics, regression, linear mixed model, statistics, statistician, statistical

## Abstract

**Background:**

Patients recovering from COVID-19 often experience persistent problems in their daily activities related to limitations in physical, nutritional, cognitive, and mental functioning. To date, it is unknown what treatment is needed to support patients in their recovery from COVID-19.

**Objective:**

This study aimed to evaluate the primary allied health care of patients recovering from COVID-19 at 6-month follow-up and to explore which baseline characteristics are associated with changes in the scores of outcomes between baseline and 6-month follow-up.

**Methods:**

This Dutch nationwide prospective cohort study evaluated the recovery of patients receiving primary allied health care (ie, dietitians, exercise therapists, occupational therapists, physical therapists, and speech and language therapists) after COVID-19. All treatments offered by primary allied health professionals in daily practice were part of usual care. Patient-reported outcome measures on participation, health-related quality of life, fatigue, physical functioning, and psychological well-being were assessed at baseline and at 3- and 6-month follow-up. Linear mixed model analyses were used to evaluate recovery over time, and uni- and multivariable linear regression analyses were used to examine the association between baseline characteristics and recovery.

**Results:**

A total of 1451 adult patients recovering from COVID-19 and receiving treatment from 1 or more primary allied health professionals were included. For participation (Utrecht Scale for Evaluation of Rehabilitation—Participation range 0-100), estimated mean differences of at least 2.3 points were observed at all time points. For the health-related quality of life (EuroQol Visual Analog Scale, range 0-100), the mean increase was 12.3 (95% CI 11.1-13.6) points at 6 months. Significant improvements were found for fatigue (Fatigue Severity Scale, range 1-7): the mean decrease was –0.7 (95% CI –0.8 to –0.6) points at 6 months. However, severe fatigue was reported by 742/929 (79.9%) patients after 6 months. For physical functioning (Patient-Reported Outcomes Measurement Information System—Physical Function Short Form 10b, range 13.8-61.3), the mean increase was 5.9 (95% CI 5.9-6.4) points at 6 months. Mean differences of –0.8 (95% CI –1.0 to –0.5) points for anxiety (Hospital Anxiety and Depression Scale range 0-21) and –1.6 (95% CI –1.8 to –1.3) points for depression were found after 6 months. A worse baseline score, hospital admission, and male sex were associated with greater improvement between baseline and 6-month follow-up, whereas age, the BMI, comorbidities, and smoking status were not associated with mean changes in any outcome measures.

**Conclusions:**

Patients recovering from COVID-19 who receive primary allied health care make progress in recovery but still experience many limitations in their daily activities after 6 months. Our findings provide reference values to health care providers and health care policy makers regarding what to expect from the recovery of patients who receive health care from 1 or more primary allied health professionals.

**Trial Registration:**

ClinicalTrials.gov NCT04735744; https://tinyurl.com/3vf337pn

**International Registered Report Identifier (IRRID):**

RR2-10.2340/jrm.v54.2506

## Introduction

An estimated 32%-57% of patients recovering from a COVID-19 infection experience severe and long-term problems in daily functioning and participation [1-3]. It is becoming increasingly clear that both patients with mild symptoms and those with serious symptoms during an acute COVID-19 infection are at risk of developing the post–COVID-19 condition [1,2,4,5]. Post–COVID-19, also referred to as “long COVID,” is defined as “signs and symptoms that develop during or after a COVID-19 infection, continuing for more than 12 weeks, and that are not explained by an alternative diagnosis” [6-8]. To date, it is unknown what treatment is needed to support patients in their recovery from COVID-19.

Patients recovering from COVID-19 often experience persistent problems in their daily activities related to limitations in physical, nutritional, cognitive, and mental functioning [3,5,9-11]. Fatigue is the most prevalent and persistent symptom, irrespective of the severity of the initial infection [3,5,10,12,13]. Longitudinal data suggest that fatigue does not resolve over time in many patients, even if they receive health care [3,9,10,13,14]. Increased levels of fatigue can result in lower levels of physical activity [15] and limit patients in activities of daily living (eg, housekeeping and grocery shopping) and outdoor pursuits [16]. Mental problems, such as anxiety and depression, are common in patients recovering from COVID-19. A study by Huang et al [17] showed that anxiety and depression were present in approximately 23% of patients 6 months after the onset of COVID-19 symptoms. Sisó-Almirall et al [10] showed that 36% of patients still reported mental problems after 3 months, and no significant associations were found with COVID-19 severity. Furthermore, previous studies have observed a worsened health-related quality of life (HRQoL) in patients recovering from COVID-19, both hospitalized and nonhospitalized, who did not recuperate after a follow-up period of several months [9,18-21].

The World Health Organization (WHO) suggests that rehabilitation for patients with the post–COVID-19 condition requires person-centered care that recommends multidisciplinary collaboration among health care professionals. These multidisciplinary rehabilitation interventions may include breathing techniques, physical exercise therapy, cognitive behavioral therapy, occupational therapy, nutritional support, and improving swallowing physiology [22]. In the Netherlands, mono- and multidisciplinary best-practice recommendations for primary allied health professionals have been developed for the treatment of patients recovering from COVID-19 [23-26]. Based on the overall effects of primary allied health care, it is expected that primary allied health professionals (ie, dietitians, exercise therapists, occupational therapists, physical therapists, and speech and language therapists) can play a role in the recovery of patients with COVID-19 who experience persistent limitations in daily physical functioning and participation. In July 2020, the Dutch Ministry of Health, Welfare and Sports instated a temporary regulation in primary allied health care to facilitate the treatment of patients recovering from COVID-19 and to stimulate research. This regulation enables the reimbursement of primary allied health care for every patient from basic health insurance coverage. With a referral from a general practitioner (GP) or medical specialist, primary allied health care treatment is reimbursed for a period of 6 months. If recovery during this period is insufficient, an extension by a second 6-month period is possible upon referral by a medical specialist. As COVID-19 is still a novel condition and the evidence base for allied health treatment in patients with post–COVID-19 syndrome is small, it is vital that new data and insights be shared as soon as they are available; therefore, the aim of this paper is to present the results of recovery of patients receiving primary allied health care after a COVID-19 infection. We provide outcomes at 3- and 6-month follow-up regarding participation, the HRQoL, physical functioning, fatigue, and psychological well-being. In addition, we explore which baseline characteristics are associated with changes in these outcomes between baseline and 6-month follow-up.

## Methods

### Study Design and Setting

As part of a nationwide project to evaluate the recovery of patients receiving primary allied health care after a COVID-19 infection, a prospective cohort study was set up in collaboration with various patient organizations (ie, the Lung Foundation Netherlands, the Netherlands Patient Federation, and Harteraad) and with input from patients contacted through these organizations [27]. In this prospective cohort study, patients were included at the start of their treatment with 1 or more primary allied health professionals. All treatments offered by primary allied health professionals in daily practice were part of usual care and were preferably based on recommendations and guidelines published by the professional bodies of the respective care providers, as available at the start of the research [23-26]. The inclusion period for the cohort study was between March 29 and June 19, 2021. Primary outcome measures were assessed at baseline (T0) and again after 3 months (T1) and 6 months (T2). The full study protocol with timelines is published elsewhere [27]. In this paper, we report the results of our primary outcome measures at baseline, 3-month follow-up, and 6-month follow-up.

### Ethical Considerations

The study protocol was approved by the Medical Ethics Committee of the Radboud University Medical Centre (registration #2020-7278). The study has been registered in the ClinicalTrials registry (NCT04735744). Informed consent was obtained from all patients before enrollment in the study, and all procedures were conducted in accordance with the Declaration of Helsinki.

### Participants

Adult patients (age≥18 years) were eligible for inclusion in the cohort if they were recovering from COVID-19 and started treatment with 1 or more primary allied health professionals (ie, a dietitian, exercise therapist, occupational therapist, physical therapist, or speech and language therapist). Patients may have received treatment from 1 or more primary allied health professionals during the course of the study. Patients were included regardless of their hospital admission status during the acute phase of COVID-19. Patients who were unable to complete questionnaires in Dutch and patients who were receiving palliative care were excluded from the study.

### Data Collection

Patients could enroll in the study by (1) signing up after an invitation from their treating primary allied health professional or (2) signing up on their own initiative, upon which the research team also invited the treating primary allied health professional to participate. The enrollment procedure of this study is described in detail in the published study protocol [27]. Both patients and primary allied health professionals reported data via the specifically designed Your Research app. Patients were asked to download the app on their smartphones or make use of the web version. Questionnaires were sent out through this app at the start of the treatment (baseline) and after 3 and 6 months. Patients unable to participate via digital methods were provided with an opportunity to complete the questionnaires on paper and return them by post. Primary allied health professionals were asked to use the web version of the app.

### Outcome Measures

Data on patient characteristics were collected by the treating primary allied health professionals at the start of the treatment. Patient-reported outcome domains (participation, HRQoL, fatigue, and physical functioning) were assessed at baseline and after 3 and 6 months. Data on psychological well-being were collected at baseline and after 6 months.

#### Patient Characteristics

Patient characteristics were collected via an online record form and contained the following items on demographics: age, sex, height (in cm), weight (in kg) both at the start of treatment and before COVID-19 infection, living status (whether the patient had an informal caregiver), and referring physician. Furthermore, data on symptom severity at the onset of treatment (ie, mild to moderate [mild symptoms up to mild pneumonia], severe [dyspnea, hypoxia, or <50% lung involvement on imaging], or critical [respiratory failure, shock, or multiorgan system dysfunction], as described in Ref. [28]) as well as hospital admissions during the acute phase of COVID-19 (ie, no hospital admission, admission to hospital ward or intensive care unit [ICU]) were recorded. Additionally, data on comorbidities (ie, cardiovascular disease, chronic lung disease, diabetes mellitus, kidney disease, liver disease, immune disease, oncological disease, chronic neuromuscular disorders) and smoking status were collected. The body weight and height were used to calculate each patient’s BMI (weight/height^2^) and categorized as defined by WHO [29].

#### Participation

Participation was assessed with the Utrecht Scale for Evaluation of Rehabilitation—Participation (USER-P). The USER-P is a 31-item self-administered questionnaire reflecting a patient’s participation in daily life, divided over 3 subscales: frequencies, restrictions, and satisfaction. The total scores range from 0 to 100 for each subscale, with higher scores indicating better participation (higher frequency, fewer restrictions, and higher satisfaction) [30]. We arbitrarily assumed a 5-point difference on 1 of these USER-P scales to be clinically relevant for patients recovering from COVID-19 [31,32].

#### Health-Related Quality of Life

The HRQoL was assessed with the EuroQoL 5 Dimensions 5 Level (EQ-5D-5L) tool, a 5-item questionnaire measuring a person’s status on 5 dimensions of health: mobility, self-care, usual activities, pain/discomfort, and anxiety/depression [33]. Furthermore, the EuroQoL Visual Analog Scale (EQ-VAS) was recorded by the patients. The EQ-VAS provides a quantitative measure of a patient’s perception of their overall health, with a score ranging from 0 to 100, with higher scores indicating a higher HRQoL. A difference of 8 points on the EQ-VAS was considered clinically relevant [34].

#### Fatigue

Fatigue was assessed with the Fatigue Severity Scale (FSS), a 9-item scale measuring the severity of fatigue and its effect on patients’ activities and lifestyle. The score of each item ranges from 1 to 7, where 1 indicates strong disagreement and 7 indicates strong agreement. The total score is calculated using the mean value of the 9 items, with a score of 4 or more indicating severe fatigue [35]. A difference of 0.45 points on the FSS mean score was considered clinically relevant [36].

#### Physical Functioning

Limitations in physical functioning were assessed with the Patient-Reported Outcomes Measurement Information System Physical Functioning Short Form 10b (PROMIS-PF-10b), a 10-item questionnaire measuring the self-reported ability to perform activities of daily life. Items reflect 4 subcategories: upper extremities (dexterity), lower extremities (walking or mobility), and central regions (neck and back), as well as instrumental activities of daily living, such as running errands [37]. Total scores range from 13.8 (severely physically impaired) to 61.3 (not physically impaired), with a mean score of 50 (SD 10) representing the mean score of a reference population [38]. A difference of 3.6 points was considered clinically relevant [39].

#### Psychological Well-Being

The Hospital Anxiety and Depression Scale (HADS) was used to assess psychological well-being. This 14-item self-administered questionnaire describes symptoms of anxiety and depression. The HADS is divided into an anxiety score (HADS-A) and a depression score (HADS-D), each containing 7 items. The total score ranges from 0 to 21 for both subscales, where a total score of 11 or more indicates a probable clinical diagnosis of depression or anxiety [40,41]. A difference of 1.7 points was considered clinically relevant [42].

### Statistical Analysis

Descriptive statistics were used to describe the patient population and to analyze the primary outcome measures at baseline and after 3 and 6 months using numbers and proportions for categorical variables, means (SDs), and medians (IQRs) for continuous variables. Linear mixed model analyses were used to evaluate recovery over time for participation, the HRQoL, fatigue, physical functioning, and psychological well-being. This analysis accounts for correlation between repeated measures on the same subject and uses all available data from this subject. A model with a random intercept and all other variables fixed was also generated. The primary outcomes were used as dependent variables, while time (categorical: baseline, 3 months, and 6 months) was used as a fixed factor.

Uni- and multivariate regression analyses were used to explore which baseline characteristics were associated with changes in the scores of the main outcome measures between baseline (T0) and 6-month follow-up (T2). This analysis used data from complete cases, and missingness at random (MAR) was tested (Table S1 in Multimedia Appendix 1). Univariate analyses were performed to determine which baseline characteristics (ie, age, sex, BMI, hospitalization, comorbidities, baseline score, and smoking status) were associated with the mean change in each outcome measure. Comorbidities were coded into 3 categories: none, 1, and 2 or more comorbidities. Variables with *P*<.157 in the univariate regression were included in the multivariate model [43]. The backward elimination of variables was then performed in order of statistical significance until only factors that were significantly associated with the outcome remained. A sensitivity analysis was performed by forcing age, sex, and hospital admission into the models as these factors are known to be related with recovery over time. Results of this sensitivity analysis are presented in Table S2 in Multimedia Appendix 1. *P*<.05 was considered statistically significant for all analyses based on 2-sided testing. All data were analyzed using SPSS Statistics version 25 (IBM Corp).

### Patient and Public Involvement

During the development of this study, we involved patients to provide feedback on the readability and appropriateness of proposed measures. The usability of the smartphone and web-based app versions was also tested by patients. Participating patients received updates on the status of the study via their smartphone or web app. Furthermore, various patient organizations (ie, the Lung Foundation Netherlands, the Netherlands Patient Federation, and Harteraad) participated during routine research meetings.

## Results

### Patient Characteristics

**Figure 1:**
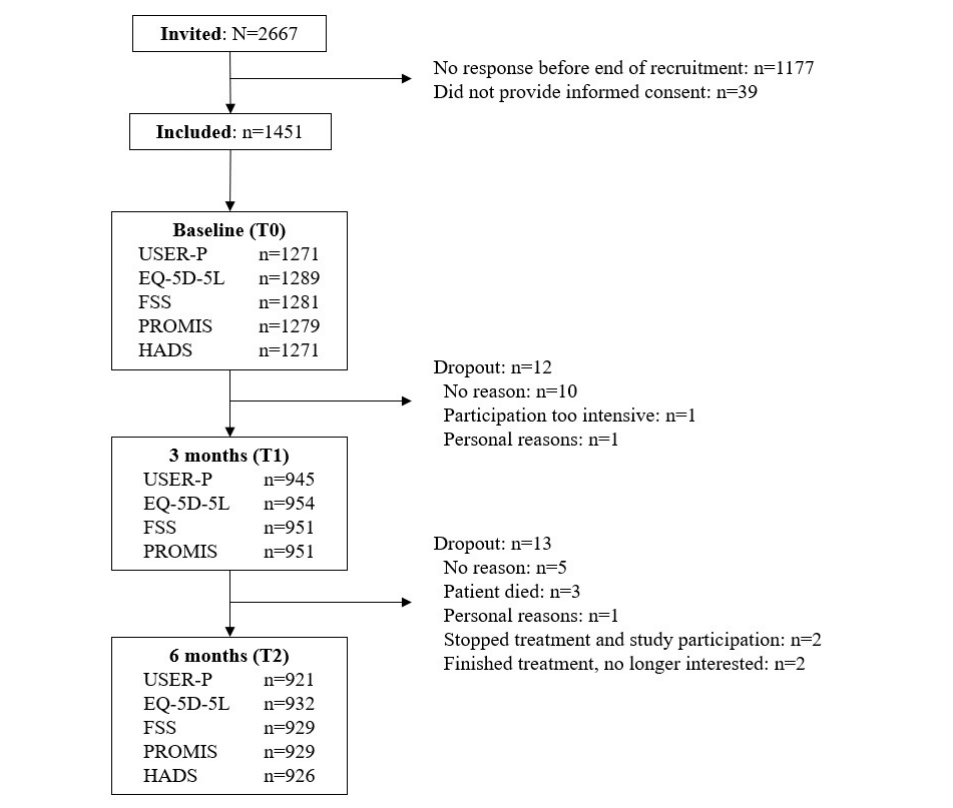
Flow diagram for inclusion of patients recovering from COVID-19 receiving primary allied health care in a Dutch prospective cohort study, with follow-up after 3 and 6 months from the start of treatment. EQ-5D-5L: EuroQoL 5 Dimensions 5 Level; FSS: Fatigue Severity Scale; HADS: Hospital Anxiety and Depression Scale; PROMIS: Patient-Reported Outcomes Measurement Information System; USER-P: Utrecht Scale for Evaluation of Rehabilitation–Participation.

In total, 1451 patients were included in this study (Figure 1), receiving 1708 different allied health care treatments. Their mean age was 49 (SD 13) years, and 63.8% (848/1330) of the patient population was female (Table 1). The majority (1015/1315, 77.2%) had not been hospitalized for COVID-19, and 1002/1311 (76.4%) patients had experienced mild-to-moderate severity of symptoms during the infection period. The mean BMI was 28 (SD 6) kg/m^2^, and 68.9% (738/1071) of the patient population was classified as being overweight or obese (BMI>25 kg/m^2^). In addition, 1 comorbidity was reported by 410/1331 (30.8%) patients, and 2 or more comorbidities were reported by 155/1331 (11.6%) patients. Cardiovascular disease (193/1331, 14.5%) and chronic lung disease (183/1331, 13.7%) were the most prevalent comorbidities. Most patients (1086/1331, 81.6%) had been referred for primary allied health care by their GP.

**Table 1:** General characteristics of patients recovering from COVID-19 receiving primary allied health care in a Dutch prospective cohort study (N=1451).

General characteristics		Participants
**Treatments (n=1708)^a^, n (%)**		
	Physical therapy/exercise therapy	1005 (58.8)
	Occupational therapy	364 (21.3)
	Dietary care	224 (13.1)
	Speech and language therapy	115 (6.7)
**Sex (n=1330)^b^, n (%)**		
	Male	482 (36.2)
	Female	848 (63.8)
Age (years; n=1331), mean (SD)		49 (13)
**COVID-19 severity (n=1311)^b^, n (%)**		
	Mild/moderate	1002 (76.4)
	Serious	271 (20.7)
	Very serious	38 (2.9)
**Admission to hospital for COVID-19 infection (n=1315)^b^, n (%)**		
	Hospitalized, including the ICU^c^	87 (6.6)
	Hospitalized	213 (16.2)
	Not hospitalized	1015 (77.2)
**BMI (kg/m^2^; n=1071)^b^; mean 28 (SD 6)**		
	Underweight (<18.5)	10 (0.9)
	Normal weight (18.5-25.0)	323 (30.2)
	Overweight (25.0-30.0)	404 (37.7)
	Obese (>30.0)	334 (31.2)
**Smoking status (n=1305)^b^, n (%)**		
	Current	63 (4.8)
	Former	166 (12.7)
	Never	1076 (82.5)
**Living status (n=1322)^b^, n (%)**		
	Alone	212 (16.0)
	Cohabiting	1110 (84.0)
**Informal caregiver (n=1319)^b^, n (%)**		
	Yes	526 (39.9)
	No	793 (60.1)
**Comorbidities (n=1331), n (%)**		
	0	766 (57.6)
	1	410 (30.8)
	≥2	155 (11.6)

^a^Some participants received multiple treatments from multiple primary allied health professionals. Therefore, the number of treatments exceeded the number of participants.

^b^Data were not fully available for all patients.

^c^ICU: intensive care unit.

### Primary Outcome Measures

Table 2 presents data on the outcome measures at baseline and at 3- and 6-month follow-up. Additionally, clinically relevant improvements at 6-month follow-up are presented in Table S3 in Multimedia Appendix 1. After 6 months, the majority of patients showed a clinically relevant improvement on the USER-P restrictions and satisfaction subscales (576/890, 64.7%, patients and 543/891, 60.9%, patients, respectively), while 540/908 (59.5%) patients showed a clinically relevant improvement on the EQ-VAS (mean 67.4, SD 19.1 points) compared to baseline (mean 55.5, SD 17.8 points). Severe fatigue was reported by 1205/1281 (94.1%) patients at baseline, persisting after 6 months in 742/929 (79.9%) patients. A clinically relevant improvement on the FSS mean score was found in 490/904 (54.2%) patients. Based on PROMIS-PF-10b scores, over two-thirds of the patients reported being more than 60% impaired, limited, or restricted in physical functioning at baseline, which decreased to 37.8% (351/929) after 6 months; 517/902 (57.3%) patients experienced a clinically relevant improvement in physical functioning. The majority of patients scored less than 7 points on the HADS anxiety and depression scores both at baseline and at 6 months, which indicates no anxiety disorder or depression. At baseline, the HADS anxiety score indicated a probable clinical diagnosis of anxiety disorder in 292/1271 (23%) patients, which decreased slightly to 170/926 (18.4%) patients after 6 months. A probable clinical diagnosis of depression was indicated by the HADS depression score in 285/1271 (22.4%) patients at baseline, decreasing to 137/926 (14.8%) patients at 6-month follow-up.

**Table 2:** General outcome measures at baseline and after 3 and 6 months in patients^a^ recovering from COVID-19 receiving primary allied health care in a Dutch prospective cohort study.

General outcome measures		Baseline (T0)	3 months (T1)	6 months (T2)
**Participation**				
	Sample population, n/N (%)	1271/1451 (87.6)	945/1451 (65.1)	921/1451 (63.5)
	USER-P^b^ frequencies subscale score, mean (SD)	27.5 (10.3)	30.5 (10.5)	31.3 (10.1)
	USER-P restrictions subscale score, mean (SD)	65.8 (20.1)	73.6 (19.4)	77.3 (19.8)
	USER-P satisfaction subscale score, mean (SD)	48.6 (17.8)	54.8 (19.5)	58.1 (19.9)
**HRQoL^c^**				
	Sample population, n/N (%)	1289/1451 (88.8)	954/1451 (65.7)	932/1451 (64.2)
	EQ-VAS^d^ score, mean (SD)	55.5 (17.8)	64.3 (18.2)	67.4 (19.1)
**Fatigue**				
	Sample population, n/N (%)	1281/1451 (88.3)	951/1451 (65.5)	929/1451 (64.0)
	FSS^e^ score, mean (SD)	5.6 (1.0)	5.2 (1.2)	4.9 (1.3)
	≥4 points, n (%)	1205 (94.1)	815 (85.7)	742 (79.9)
**Physical functioning**				
	Sample population, n/N (%)	1279/1451 (88.1)	951/1451 (65.5)	929/1451 (64.0)
	PROMIS-PF-10b^f^ score, mean (SD)	37.7 (6.0)	41.5 (7.6)	43.5 (8.50)
	100% impaired, limited, or restricted, n (%)	2 (0.2)	2 (0.2)	140 (15.1)
	80%-99% impaired, limited, or restricted, n (%)	427 (33.4)	176 (18.5)	211 (22.7)
	60%-79% impaired, limited, or restricted, n (%)	452 (35.3)	276 (29.0)	202 (21.7)
	40%-59% impaired, limited, or restricted, n (%)	259 (20.3)	227 (23.9)	157 (16.9)
	20%-39% impaired, limited, or restricted, n (%)	97 (7.6)	133 (14.0)	150 (16.1)
	1%-19% impaired, limited, or restricted, n (%)	39 (3.0)	95 (10.0)	72 (7.8)
	0% impaired, limited, or restricted, n (%)	4 (0.3)	42 (4.4)	140 (15.1)
**Psychological well-being^g^ (anxiety)**				
	Sample population, n/N (%)	1271/1451 (87.6)	N/A^h^	926/1451 (63.8)
	HADS^i^ anxiety score, mean (SD)	7.1 (4.5)	N/A	6.3 (4.7)
	≤7 points, n (%)	746 (58.7)	N/A	613 (66.2)
	8-10 points, n (%)	233 (18.3)	N/A	143 (15.4)
	≥11 points, n (%)	292 (23.0)	N/A	170 (18.4)
**Psychological well-being (depression)**				
	Sample population, n/N (%)	1271/1451 (87.6)	N/A	926/1451 (63.8)
	HADS depression score, mean (SD)	7.3 (4.2)	N/A	5.7 (4.3)
	≤7 points, n (%)	689 (54.2)	N/A	638 (68.9)
	8-10 points, n (%)	297 (23.4)	N/A	151 (16.3)
	≥11 points, n (%)	285 (22.4)	N/A	137 (14.8)

^a^Data were not fully available for all patients.

^b^USER-P: Utrecht Scale for Evaluation of Rehabilitation—Participation.

^c^HRQoL: health-related quality of life.

^d^EQ-VAS: EuroQol Visual Analog Scale.

^e^FSS: Fatigue Severity Scale.

^f^PROMIS-PF-10b: Patient-Reported Outcomes Measurement Information System Physical Functioning Short Form 10b.

^g^Psychological well-being was only assessed at baseline (T0) and at 6 months (T2).

^h^N/A: not applicable.

^i^HADS: Hospital Anxiety and Depression Scale.

### Patient-Reported Recovery Over Time

Table 3 shows the effect of time on the outcome measures. For all dependent variables, a random intercept model was the best-fitting model. No variables were significantly related to missing values in the outcome measures at any point in time. A significant effect of time was observed for all outcome measures at 3- and 6-month follow-up (*P*<.001). For participation, estimated mean differences of at least 2.9 points (*P*<.001) were observed for all 3 subscales at all time points. For the HRQoL, the mean increase was 9.0 points (95% CI 7.8-10.2) at 3 months and 12.3 points (95% CI 11.1-13.6) after 6 months. Furthermore, significant improvements were found for fatigue and physical functioning at all time points. The greatest improvements were seen after just 3 months for all outcome measures measured at both 3 and 6 months. Mean differences of –0.8 (95% CI –1.0 to –0.5) on the HADS anxiety score and –1.6 (95% CI –1.8 to –1.3) on the HADS depression score were observed.

**Table 3:** Results of linear mixed model analysis for the outcome measures participation, HRQoL^a^, fatigue, physical functioning, and psychological well-being in patients recovering from COVID-19 receiving primary allied health care in a Dutch prospective cohort study.

General outcome measures			Baseline (T0), mean (SE)		3 months (T1), mean (SE)		6 months (T2), mean (SE)		At 3 months						At 6 months				
										Estimate (95% CI)			*P* value			Estimate (95% CI)			*P* value
**Participation**																			
	USER-P^b^ frequencies subscale	27.5 (0.3)		30.5 (0.3)		31.5 (0.3)		2.9 (2.3 to 3.7)			<.001			3.9 (3.3 to 4.7)			<.001		
	USER-P restrictions subscale	64.6 (0.6)		73.7 (0.6)		77.6 (0.6)		9.1 (7.9 to 10.3)			<.001			13.0 (11.8 to 14.2)			<.001		
	USER-P satisfaction subscale	48.7 (0.5)		54.7 (0.6)		58.4 (0.6)		5.9 (4.8 to 7.2)			<.001			9.7 (8.5 to 10.9)			<.001		
HRQoL (EQ-VAS^c^ score)			55.6 (0.5)		64.6 (0.6)		67.9 (0.7)		9.0 (7.8 to 10.2)			<.001		12.3 (11.1 to 13.6)				<.001	
Fatigue (FSS^d^ score)			5.6 (0.03)		5.2 (0.04)		4.9 (0.04)		–0.4 (–0.5 to –0.4)			<.001		–0.7 (–0.8 to –0.6)				<.001	
Physical functioning (PROMIS-PF-10b^e^ score)			37.7 (0.2)		41.6 (0.2)		43.7 (0.2)		3.9 (3.5 to 4.3)			<.001		5.9 (5.6 to 6.4)				<.001	
**Psychological well-being^f^**																			
	HADS^g^ anxiety score	7.1 (0.1)		N/A^h^		6.3 (0.1)		N/A			N/A			–0.8 (–1.0 to –0.5)			<.001		
	HADS depression score	7.3 (0.1)		N/A		5.7 (0.1)		N/A			N/A			–1.6 (–1.8 to –1.3)			<.001		

^a^HRQoL: health-related quality of life.

^b^USER-P: Utrecht Scale for Evaluation of Rehabilitation—Participation.

^c^EQ-VAS: EuroQol Visual Analog Scale.

^d^FSS: Fatigue Severity Scale.

^e^PROMIS-PF-10b: Patient-Reported Outcomes Measurement Information System Physical Functioning Short Form 10b.

^f^Psychological well-being was only assessed at baseline (T0) and at 6 months (T2).

^g^HADS: Hospital Anxiety and Depression Scale.

^h^N/A: not applicable.

### Factors Associated With Changes in the Scores of the Main Outcome Measures

Multivariable regression models were estimated to identify factors associated with changes in scores between baseline and 6-month follow-up for each outcome measure. Tables 4-10 provide an overview of the final regression models. All univariable and multivariable regression models are shown in Tables S4 and S5 in Multimedia Appendix 1. Having a worse baseline score was related to greater improvements for all outcome measures. For all 3 subscales of the USER-P and physical functioning, patients admitted to the hospital during the infection period of COVID-19 showed greater improvements in scores than nonhospitalized patients, even when correcting for baseline scores. In terms of the HRQoL, patients admitted to a hospital ward showed greater improvements than patients who had not been hospitalized, although no associations were found with ICU admissions. Male participants showed greater improvements than female participants in all outcome measures, except for psychological well-being, for which no association was found for sex. The baseline age, BMI, comorbidities, and smoking status were not significantly associated with the mean change in any of the outcome measures in our patient population. In a sensitivity analysis (Table S2 in Multimedia Appendix 1) where age, sex, and hospital admission were forced into the model, additional associations were found between the male sex and satisfaction in participation and between age and frequencies of participation, physical functioning, and symptoms of anxiety.

**Table 4:** Multivariable linear regression models on the outcome measure participation (USER-P^a^ frequencies subscale) in patients recovering from COVID-19 receiving primary allied health care in a Dutch prospective cohort study (R2 overall model=0.272, *P*<.001).

Outcome measure			β	95% CI		*P* value
**Hospital admission**						.001
	No	Reference		N/A^b^	N/A	
	Hospital ward	2.556		0.851 to 4.262	.003	
	ICU^c^	3.079		0.611 to 5.547	.015	
Baseline score			–.496	–0.558 to –0.435		<.001

^a^USER-P: Utrecht Scale for Evaluation of Rehabilitation—Participation.

^b^N/A: not applicable.

^c^ICU: intensive care unit.

**Table 5:** Multivariable linear regression models on the outcome measure participation (USER-P^a^ restrictions subscale) in patients recovering from COVID-19 receiving primary allied health care in a Dutch prospective cohort study (R2 overall model=0.277, *P*<.001).

Outcome measure		β	95% CI	*P* value	
**Sex**					.001
	Male	Reference	N/A^b^	N/A	
	Female	–5.337	–7.813 to –2.861	.001	
**Hospital admission**					<.001
	No	Reference	N/A	N/A	
	Hospital ward	3.581	0.316 to 6.845	.032	
	ICU^c^	9.165	4.522 to 13.809	<.001	
Baseline score		–.462	–0.520 to –0.405	<.001	

^a^USER-P: Utrecht Scale for Evaluation of Rehabilitation—Participation.

^b^N/A: not applicable.

^c^ICU: intensive care unit.

**Table 6:** Multivariable linear regression models on the outcome measure participation (USER-P^a^ satisfaction subscale) in patients recovering from COVID-19 receiving primary allied health care in a Dutch prospective cohort study (R2 overall model=0.159, *P*<.001).

Outcome measure			β	95% CI		*P* value
**Hospital admission**						.003
	No	Reference		N/A^b^	N/A	
	Hospital ward	3.577		0.356 to 6.798	.030	
	ICU^c^	6.728		2.144 to 11.311	.004	
Baseline score			–.402	–0.467 to –0.338		<.001

^a^USER-P: Utrecht Scale for Evaluation of Rehabilitation—Participation.

^b^N/A: not applicable.

^c^ICU: intensive care unit.

**Table 7:** Multivariable linear regression models on the outcome measure HRQoL^a^ (EQ-VAS^b^ score) in patients recovering from COVID-19 receiving primary allied health care in a Dutch prospective cohort study (R2 overall model=0.245, *P*<.001).

Outcome measure			β	95% CI		*P* value
**Sex**						<.001
	Male	Reference		N/A^c^	N/A	
	Female	–4.855		–7.378 to –2.333	<.001	
**Hospital admission**						.10
	No	Reference		N/A	N/A	
	Hospital ward	3.594		0.231 to 6.957	.036	
	ICU^d^	2.106		–2.615 to 6.827	.38	
Baseline score			–.524	–0.589 to –0.459		<.001

^a^HRQoL: health-related quality of life.

^b^EQ-VAS: EuroQol Visual Analog Scale.

^c^N/A: not applicable.

^d^ICU: intensive care unit.

**Table 8:** Multivariable linear regression models on the outcome measure fatigue (FSS^a^ score) in patients recovering from COVID-19 receiving primary allied health care in a Dutch prospective cohort study (R2 overall model=0.064, *P*<.001).

Outcome measure			β	95% CI		*P* value
**Sex**						<.001
	Male	Reference		N/A^b^	N/A	
	Female	.284		0.130 to 0.438	<.001	
Baseline score			–.301	–0.381 to –0.222		<.001

^a^FSS: Fatigue Severity Scale.

^b^N/A: not applicable.

**Table 9:** Multivariable linear regression models on the outcome measure physical functioning (PROMIS-PF-10b^a^ score) in patients recovering from COVID-19 receiving primary allied health care in a Dutch prospective cohort study (R2 overall model=0.064, *P*<.001).

Outcome measure			β	95% CI		*P* value
**Sex**						<.001
	Male	Reference		N/A^b^	N/A	
	Female	–2.342		–3.341 to –1.343	<.001	
**Hospital admission**						
	No	Reference		N/A	.004	
	Hospital ward	1.149		–0.165 to 2.463	.09	
	ICU^c^	2.917		1.064 to 4.771	.002	
Baseline score			–.125	–0.203 to –0.046		<.001

^a^PROMIS-PF-10b: Patient-Reported Outcomes Measurement Information System Physical Functioning Short Form 10b.

^b^N/A: not applicable.

^c^ICU: intensive care unit.

**Table 10:** Multivariable linear regression models on the outcome measure psychological well-being (HADS^a^ anxiety and depression scores; all baseline scores) in patients recovering from COVID-19 receiving primary allied health care in a Dutch prospective cohort study.

Outcome measure	β	R^2^ overall model	95% CI	*P* value
HADS anxiety	–.354	0.160 (*P*<.001)	–0.407 to –0.301	<.001
HADS depression	–.392	0.179 (*P*<.001)	–0.447 to –0.337	<.001

^a^HADS: Hospital Anxiety and Depression Scale.

## Discussion

### Principal Findings

This study presents the first results of our evaluation of the recovery of our unique cohort of patients with COVID-19 receiving primary allied health care until their 6-month follow-up. We explored which baseline characteristics were associated with changes in the scores of the main outcome measures over this 6-month period. Most patients showed a clinically relevant improvement in all outcome measures; however, despite improvement, many patients still experienced persistent problems in their daily lives, with limitations in physical and mental functioning. A worse baseline score, hospital admission, and, for some outcome measures, the male sex were associated with greater improvement between baseline and 6-month follow-up; however, age, the BMI, comorbidities, and smoking status were not associated with the mean change in any of the outcome measures.

### Comparison With Other Studies

The majority of our patient population showed a clinically relevant improvement 6 months after starting treatment provided by 1 or more primary allied health professionals; nevertheless, a large group of patients experienced persistent problems in their daily lives. The mean EQ-VAS score of our patient population (67 points) remained well below the population norm in the Netherlands, which is 82 points [44]. These results are consistent with previous findings that the HRQoL is impaired in the majority of patients post–COVID-19 [12,15,17,45-48]. Persistent fatigue was highly prevalent among the patients included in our study, with 79.9% still reporting severe fatigue (measured with the FSS) after 6 months. These results are consistent with previous studies on patients recovering from COVID-19, showing that fatigue is the most common complaint [5,10,14,47,49-51], even after 6 months [13,15,52-54]. The mean PROMIS-PF-10b score of our population (mean 43.5, SD 8.5) remained well below the population norm in the Netherlands (mean 50, SD 10). These results are also consistent with previous studies [15,47] and indicate that persistent symptoms due to COVID-19 may lead to patients experiencing limitations in physical functioning.

Relative to other outcome measures, a smaller percentage of patients showed a clinically relevant improvement in psychological well-being. This was due to an observed ceiling effect, as 58.7% and 54.2% of patients showed no indication of an anxiety disorder or depression at baseline, respectively. Data of these patients are still informative, however, as they could also have deteriorated throughout the follow-up period. With scores indicating a probable clinical diagnosis of anxiety disorder or depression in 18.4% and 14.8% of patients, respectively, after 6 months, our findings are similar to those reported in previous studies, which showed prevalence rates ranging from 11% to 40% [8,17,46,48,51,54-57]. Furthermore, we performed an additional subgroup analysis to explore the differences in changes in the scores of the outcome measures between patients who showed indications of depression or anxiety disorder at baseline and patients who did not (data not shown). Based on this analysis, we conclude that whether a patient shows indications of depression or anxiety disorder at baseline has little effect on the change in their scores over time.

We found that male participants showed greater improvements than female participants in participation, the HRQoL, fatigue, and physical functioning. These results are consistent with previous studies showing that female participants experience more persistent symptoms after a COVID-19 infection [10,14,21,45,49,51]. Furthermore, patients admitted to the hospital for COVID-19 showed greater improvements than nonhospitalized patients in terms of participation, the HRQoL, and physical functioning, which is in line with previous studies [12,21,45,51]. We observed no associations between fatigue and hospital admission, age, the BMI, comorbidities, or smoking status, which is also consistent with other studies [10,14,51,53,54], indicating that fatigue is highly prevalent in patients recovering from COVID-19, irrespective of the severity of initial infection and patient characteristics. We found that having a worse baseline score is related to greater improvement in anxiety and depressive symptoms; however, no associations with any patient characteristics were found. Similar to our results, previous studies have found no associations between the frequency of anxiety or depressive symptoms and disease severity or hospital admission [10,46,48,49,57]. In contrast, other studies have found the female sex [54,58,59] and older age [54,56] to be predictors of anxiety or depressive symptoms in patients with COVID-19. Although the association between age and symptoms of anxiety was not evident in our primary analysis, it did show up in our sensitivity analysis (Table S2 in Multimedia Appendix 1).

### Limitations

This study did not include a control group to determine the potential effects of primary allied health care by comparing outcome measures with patients who did not receive this type of care. In addition, with a lack of available pre–COVID-19 data for our population, it was difficult to draw conclusions about the impact of pre-existing conditions versus problems in the daily activities and participation of these patients due to their COVID-19 infection.

For the interpretation of results, it is important to consider that the baseline measurement in this study was taken at the start of the treatment by 1 or more primary allied health professionals. It is possible that a patient had already experienced symptoms for some time and only consulted a primary allied health professional at a later stage. Additionally, it should be considered that not all patients received treatment from 1 or more primary allied health professionals during the entire 6-month follow-up period of this study. Some patients received short-term treatment, while others were still receiving treatment at 6 months.

The characteristics of included patients are comparable to COVID-19 populations described by others [3], and therefore, we assume that the study population is representative of the population in 2021, our inclusion period. Based on the inclusion period, which was between March and July 2021, our population most likely had the Wuhan or Alpha variant of SARS-CoV-2 [60]. Different variants may cause different symptoms, and the recovery of patients infected with other variants (eg, Delta or Omicron) may differ from that of our population. A total of 25 patients dropped out during this study (Figure 1). Although a proportion of the patients did not complete all questionnaires, the response rates were still sufficient: 93% at baseline, 68% after 3 months, and 67% after 6 months [61]. There was no selective missingness of data based on patient characteristics (including disease severity) and scores of the outcome measures (Table S1 in Multimedia Appendix 1).

### Implications and Future Perspectives

Future research and in-depth analyses of our data are needed to gain more insight into the outcome measures and recovery of patients after COVID-19 who visit 1 or more primary allied health professionals. Future papers will include the results after a 12-month follow-up, determining the related health care costs and profession-specific outcomes per allied health discipline.

### Conclusion

The results of this study show that patients recovering from COVID-19 and receiving primary allied health care make progress in recovery, but many still experience limitations in their daily activities and participation after 6 months. The findings of our study provide reference values for health care providers and health care policy makers about what to expect from the recovery of patients who receive or have received health care from 1 or more primary allied health professionals.

## Appendix

Multimedia Appendix 1 Supplementary tables.

## Abbreviations

EQ-5D-5L: EuroQoL 5 Dimensions 5 Level
EQ-VAS: EuroQol Visual Analog Scale
FSS: Fatigue Severity Scale
HADS: Hospital Anxiety and Depression Scale
HRQoL: health-related quality of life
ICU: intensive care unit
PROMIS-PF-10b: Patient-Reported Outcomes Measurement Information System Physical Functioning Short Form 10b
USER-P: Utrecht Scale for Evaluation of Rehabilitation—Participation

## Data Availability

The data sets generated and analyzed during this study are available from the corresponding author upon reasonable request.

## References

[1] Taquet M, Dercon Q, Luciano S, Geddes JR, Husain M, Harrison PJ (2021). Incidence, co-occurrence, and evolution of long-COVID features: a 6-month retrospective cohort study of 273,618 survivors of COVID-19. PLoS Med.

[2] Webber S, Tittlemier BJ, Loewen Hal J (2021). Apparent discordance between the epidemiology of COVID-19 and recommended outcomes and treatments: a scoping review. Phys Ther.

[3] Ballering AV, van Zon SKR, olde Hartman TC, Rosmalen JGM (2022). Persistence of somatic symptoms after COVID-19 in the Netherlands: an observational cohort study. Lancet.

[4] Augustin M, Schommers P, Stecher M, Dewald F, Gieselmann L, Gruell H, Horn C, Vanshylla K, Cristanziano VD, Osebold L, Roventa M, Riaz T, Tschernoster N, Altmueller J, Rose L, Salomon S, Priesner V, Luers JC, Albus C, Rosenkranz S, Gathof B, Fätkenheuer G, Hallek M, Klein F, Suárez I, Lehmann C (2021). Post-COVID syndrome in non-hospitalised patients with COVID-19: a longitudinal prospective cohort study. Lancet Reg Health Eur.

[5] Goërtz YMJ, Van Herck M, Delbressine JM, Vaes AW, Meys R, Machado FVC, Houben-Wilke S, Burtin C, Posthuma R, Franssen FME, van Loon N, Hajian B, Spies Y, Vijlbrief H, van 't Hul AJ, Janssen DJA, Spruit MA (2020). Persistent symptoms 3 months after a SARS-CoV-2 infection: the post-COVID-19 syndrome?. ERJ Open Res.

[6] (2020). COVID-19 rapid guideline: managing the long-term effects of COVID-19. National Institute for Health and Care Excellence.

[7] Shah W, Hillman T, Playford ED, Hishmeh L (2021). Managing the long term effects of covid-19: summary of NICE, SIGN, and RCGP rapid guideline. BMJ.

[8] Nalbandian A, Sehgal K, Gupta A, Madhavan MV, McGroder C, Stevens JS, Cook JR, Nordvig AS, Shalev D, Sehrawat TS, Ahluwalia N, Bikdeli B, Dietz D, Der-Nigoghossian C, Liyanage-Don N, Rosner GF, Bernstein EJ, Mohan S, Beckley AA, Seres DS, Choueiri TK, Uriel N, Ausiello JC, Accili D, Freedberg DE, Baldwin M, Schwartz A, Brodie D, Garcia CK, Elkind MSV, Connors JM, Bilezikian JP, Landry DW, Wan EY (2021). Post-acute COVID-19 syndrome. Nat Med.

[9] Carfì A, Bernabei R, Landi F, Gemelli Against COVID-19 Post-Acute Care Study Group (2020). Persistent symptoms in patients after acute COVID-19. JAMA.

[10] Sisó-Almirall A, Brito-Zerón P, Conangla Ferrín L, Kostov B, Moragas Moreno A, Mestres J, Sellarès J, Galindo G, Morera R, Basora J, Trilla A, Ramos-Casals M (2021). Long Covid-19: proposed primary care clinical guidelines for diagnosis and disease management. Int J Environ Res Public Health.

[11] Maxwell E (2020). Living with Covid19. National Institute for Health Research.

[12] Malik P, Patel K, Pinto C, Jaiswal R, Tirupathi R, Pillai S, Patel U (2022). Post-acute COVID-19 syndrome (PCS) and health-related quality of life (HRQoL)—a systematic review and meta-analysis. J Med Virol.

[13] Van Herck M, Goërtz YMJ, Houben-Wilke S, Machado FVC, Meys R, Delbressine JM, Vaes AW, Burtin C, Posthuma R, Franssen FME, Hajian B, Vijlbrief H, Spies Y, van 't Hul AJ, Janssen DJA, Spruit MA (2021). Severe fatigue in long COVID: web-based quantitative follow-up study in members of online long COVID support groups. J Med Internet Res.

[14] Townsend L, Dyer AH, Jones K, Dunne J, Mooney A, Gaffney F, O'Connor L, Leavy D, O'Brien K, Dowds J, Sugrue JA, Hopkins D, Martin-Loeches I, Ni Cheallaigh C, Nadarajan P, McLaughlin AM, Bourke NM, Bergin C, O'Farrelly C, Bannan C, Conlon N (2020). Persistent fatigue following SARS-CoV-2 infection is common and independent of severity of initial infection. PLoS One.

[15] Tabacof L, Tosto-Mancuso J, Wood J, Cortes M, Kontorovich A, McCarthy D, Rizk D, Rozanski G, Breyman E, Nasr L, Kellner C, Herrera JE, Putrino D (2022). Post-acute COVID-19 syndrome negatively impacts physical function, cognitive function, health-related quality of life, and participation. Am J Phys Med Rehabil.

[16] Humphreys H, Kilby L, Kudiersky N, Copeland R (2021). Long COVID and the role of physical activity: a qualitative study. BMJ Open.

[17] Huang C, Li X, Gu X, Zhang H, Ren L, Guo Li, Liu M, Wang Y, Cui D, Wang Y, Zhang X, Shang L, Zhong J, Wang X, Wang J, Cao B (2022). Health outcomes in people 2 years after surviving hospitalisation with COVID-19: a longitudinal cohort study. Lancet Respir Med.

[18] Nguyen HC, Nguyen MH, Do BN, Tran CQ, Nguyen TTP, Pham KM, Pham LV, Tran KV, Duong TT, Tran TV, Duong TH, Nguyen TT, Nguyen QH, Hoang TM, Nguyen KT, Pham TTM, Yang S, Chao JC, Duong TV (2020). People with suspected COVID-19 symptoms were more likely depressed and had lower health-related quality of life: the potential benefit of health literacy. J Clin Med.

[19] Zhang Y, Ma ZF (2020). Impact of the COVID-19 pandemic on mental health and quality of life among local residents in Liaoning Province, China: a cross-sectional study. Int J Environ Res Public Health.

[20] Qu G, Zhen Q, Wang W, Fan S, Wu Q, Zhang C, Li B, Liu G, Yu Y, Li Y, Yong L, Lu B, Ding Z, Ge H, Mao Y, Chen W, Xu Q, Zhang R, Cao L, Chen S, Li H, Zhang H, Hu X, Zhang J, Wang Y, Zhang H, Liang C, Sun L, Sun Y (2021). Health-related quality of life of COVID-19 patients after discharge: a multicenter follow-up study. J Clin Nurs.

[21] Arab-Zozani M, Hashemi F, Safari H, Yousefi M, Ameri H (2020). Health-related quality of life and its associated factors in COVID-19 patients. Osong Public Health Res Perspect.

[22] (2023). Clinical management of COVID-19. World Health Organization.

[23] (2020). KNGF-standpunt Fysiotherapie bij COVID-19: aanbevelingen voor fysiotherapeutisch handelen tijdens de ziekenhuisopname. Royal Dutch Society for Physical Therapy (KNGF).

[24] (2020). Position statment COVID-19 version 1.4. Dutch Society for Speech-Language Therapy (NVLF).

[25] (2021). Handreiking ergotherapie bij COVID-19 cliënten in de herstelfase. Dutch Society for Occupational Therapy (EN).

[26] (2021). Treatment plan of dietitian at COVID-19 after hospital discharge. The recovery phase after discharge from hospital: dietetics treatment in (Post) COVID-19 patients in a rehabilitation center. Dutch Society for Dietitians (NVD).

[27] De Bie RA, Verburg AC, Agasi-Idenburg C, Cup EHC, Dekker C, Van Dongen JM, Geleijn E, Gerards MHG, Graff M, Van Heerde R, Kalf H, Kammerer M, Kool RA, De Kruif A, Kruizenga HM, Van der Leeden M, Lenssen TAF, Meijer WM, Ostelo R, Ronteltap A, Van der Schaaf M, Van Oers S, De van der Schueren MAE, Slotegraaf AI, Veenhof C, Hoogeboom TJ, Van der Wees P (2022). Evaluation of allied healthcare in patients recovering from Covid-19: study protocol and baseline data of a national prospective cohort study. J Rehabil Med.

[28] (2022). Interim clinical guidance for management of patients with confirmed coronavirus disease (COVID-19). Centers for Disease Control and Prevention.

[29] (2010). Body mass index - BMI. World Health Organization.

[30] Post MWM, van der Zee CH, Hennink J, Schafrat CG, Visser-Meily JM, van Berlekom SB (2012). Validity of the Utrecht Scale for Evaluation of Rehabilitation-Participation. Disabil Rehabil.

[31] van der Zee CH, Baars-Elsinga A, Visser-Meily JM, Post MW (2013). Responsiveness of two participation measures in an outpatient rehabilitation setting. Scand J Occup Ther.

[32] van der Zee CH, Kap A, Rambaran Mishre R, Schouten EJ, Post MWM (2011). Responsiveness of four participation measures to changes during and after outpatient rehabilitation. J Rehabil Med.

[33] Herdman M, Gudex C, Lloyd A, Janssen M, Kind P, Parkin D, Bonsel G, Badia X (2011). Development and preliminary testing of the new five-level version of EQ-5D (EQ-5D-5L). Qual Life Res.

[34] Zanini A, Aiello M, Adamo D, Casale S, Cherubino F, Della Patrona S, Raimondi E, Zampogna E, Chetta A, Spanevello A (2015). Estimation of minimal clinically important difference in EQ-5D Visual Analog Scale score after pulmonary rehabilitation in subjects with COPD. Respir Care.

[35] Valko P, Bassetti CL, Bloch KE, Held U, Baumann CR (2008). Validation of the Fatigue Severity Scale in a Swiss cohort. Sleep.

[36] Rooney S, McFadyen DA, Wood DL, Moffat DF, Paul PL (2019). Minimally important difference of the Fatigue Severity Scale and modified Fatigue Impact Scale in people with multiple sclerosis. Mult Scler Relat Disord.

[37] Cella D, Yount S, Rothrock N, Gershon R, Cook K, Reeve B, Ader D, Fries JF, Bruce B, Rose M, PROMIS Cooperative Group (2007). The Patient-Reported Outcomes Measurement Information System (PROMIS): progress of an NIH Roadmap cooperative group during its first two years. Med Care.

[38] Terwee CB, Roorda LD, de Vet HCW, Dekker J, Westhovens R, van Leeuwen J, Cella D, Correia H, Arnold B, Perez B, Boers M (2014). Dutch–Flemish translation of 17 item banks from the Patient-Reported Outcomes Measurement Information System (PROMIS). Qual Life Res.

[39] Sandvall B, Okoroafor UC, Gerull W, Guattery J, Calfee RP (2019). Minimal clinically important difference for PROMIS physical function in patients with distal radius fractures. J Hand Surg Am.

[40] Snaith RP (2003). The Hospital Anxiety and Depression Scale. Health Qual Life Outcomes.

[41] Zigmond AS, Snaith RP (1983). The Hospital Anxiety and Depression Scale. Acta Psychiatr Scand.

[42] Lemay K, Tulloch HE, Pipe AL, Reed JL (2019). Establishing the minimal clinically important difference for the Hospital Anxiety and Depression Scale in patients with cardiovascular disease. J Cardiopulm Rehabil Prev.

[43] Steyerberg E (2009). Clinical Prediction Models: A Practical Approach to Development, Validation, and Updating.

[44] Janssen B, Szende A, Szende A, Janssen B, Cabases J (2014). Population norms for the EQ-5D. Self-Reported Population Health: An International Perspective based on EQ-5D.

[45] Garrigues E, Janvier P, Kherabi Y, Le Bot A, Hamon A, Gouze H, Doucet L, Berkani S, Oliosi E, Mallart E, Corre F, Zarrouk V, Moyer J, Galy A, Honsel V, Fantin B, Nguyen Y (2020). Post-discharge persistent symptoms and health-related quality of life after hospitalization for COVID-19. J Infect.

[46] Rass V, Beer R, Schiefecker AJ, Kofler M, Lindner A, Mahlknecht P, Heim B, Limmert V, Sahanic S, Pizzini A, Sonnweber T, Tancevski I, Scherfler C, Zamarian L, Bellmann-Weiler R, Weiss G, Djamshidian A, Kiechl S, Seppi K, Loeffler-Ragg J, Pfausler B, Helbok R (2021). Neurological outcome and quality of life 3 months after COVID-19: a prospective observational cohort study. Eur J Neurol.

[47] Vaes AW, Goërtz YMJ, Van Herck M, Machado FV, Meys R, Delbressine JM, Houben-Wilke S, Gaffron S, Maier D, Burtin C, Posthuma R, van Loon NP, Franssen FM, Hajian B, Simons SO, van Boven JF, Klok FA, Spaetgens B, Pinxt CM, Liu LY, Wesseling G, Spies Y, Vijlbrief H, van 't Hul AJ, Janssen DJ, Spruit MA (2021). Recovery from COVID-19: a sprint or marathon? 6-Month follow-up data from online long COVID-19 support group members. ERJ Open Res.

[48] van den Borst B, Peters JB, Brink M, Schoon Y, Bleeker-Rovers CP, Schers H, van Hees HWH, van Helvoort H, van den Boogaard M, van der Hoeven H, Reijers MH, Prokop M, Vercoulen J, van den Heuvel M (2021). Comprehensive health assessment 3 months after recovery from acute coronavirus disease 2019 (COVID-19). Clin Infect Dis.

[49] Ceban F, Ling S, Lui LM, Lee Y, Gill H, Teopiz KM, Rodrigues NB, Subramaniapillai M, Di Vincenzo JD, Cao B, Lin K, Mansur RB, Ho RC, Rosenblat JD, Miskowiak KW, Vinberg M, Maletic V, McIntyre RS (2022). Fatigue and cognitive impairment in post-COVID-19 syndrome: a systematic review and meta-analysis. Brain Behav Immun.

[50] Pavli A, Theodoridou M, Maltezou HC (2021). Post-COVID syndrome: Incidence, clinical spectrum, and challenges for primary healthcare professionals. Arch Med Res.

[51] Shanbehzadeh S, Tavahomi M, Zanjari N, Ebrahimi-Takamjani I, Amiri-Arimi S (2021). Physical and mental health complications post-COVID-19: scoping review. J Psychosom Res.

[52] Alkodaymi MS, Omrani OA, Fawzy NA, Shaar BA, Almamlouk R, Riaz M, Obeidat M, Obeidat Y, Gerberi D, Taha RM, Kashour Z, Kashour T, Berbari EF, Alkattan K, Tleyjeh IM (2022). Prevalence of post-acute COVID-19 syndrome symptoms at different follow-up periods: a systematic review and meta-analysis. Clin Microbiol Infect.

[53] Ezzat MM, MD DAE, Elsherif AA (2021). Prevalence of fatigue in patients post COVID-19. Eur J Mol Clin Med.

[54] Menges D, Ballouz T, Anagnostopoulos A, Aschmann HE, Domenghino A, Fehr JS, Puhan MA (2021). Burden of post-COVID-19 syndrome and implications for healthcare service planning: A population-based cohort study. PLoS One.

[55] González J, Benítez ID, Carmona P, Santisteve S, Monge A, Moncusí-Moix A, Gort-Paniello C, Pinilla L, Carratalá A, Zuil M, Ferrer R, Ceccato A, Fernández L, Motos A, Riera J, Menéndez R, Garcia-Gasulla D, Peñuelas O, Bermejo-Martin JF, Labarca G, Caballero J, Torres G, de Gonzalo-Calvo D, Torres A, Barbé F, CIBERESUCICOVID Project (COV20/00110‚ ISCIII) (2021). Pulmonary function and radiologic features in survivors of critical COVID-19: a 3-month prospective cohort. Chest.

[56] Morin L, Savale L, Pham T, Colle R, Figueiredo S, Harrois A, Gasnier M, Lecoq A-L, Meyrignac O, Noel N, Baudry E, Bellin M-F, Beurnier A, Choucha W, Corruble E, Dortet L, Hardy-Leger I, Radiguer F, Sportouch S, Verny C, Wyplosz B, Zaidan M, Becquemont L, Montani D, Monnet X, Writing Committee for the COMEBAC Study Group (2021). Four-month clinical status of a cohort of patients after hospitalization for COVID-19. JAMA.

[57] Renaud-Charest O, Lui LM, Eskander S, Ceban F, Ho R, Di Vincenzo JD, Rosenblat JD, Lee Y, Subramaniapillai M, McIntyre RS (2021). Onset and frequency of depression in post-COVID-19 syndrome: a systematic review. J Psychiatr Res.

[58] Mazza MG, Palladini M, De Lorenzo R, Magnaghi C, Poletti S, Furlan R, Ciceri F, Rovere-Querini P, Benedetti F, COVID-19 BioB Outpatient Clinic Study Group (2021). Persistent psychopathology and neurocognitive impairment in COVID-19 survivors: effect of inflammatory biomarkers at three-month follow-up. Brain Behav Immun.

[59] Righi E, Mirandola M, Mazzaferri F, Dossi G, Razzaboni E, Zaffagnini A, Ivaldi F, Visentin A, Lambertenghi L, Arena C, Micheletto C, Gibellini D, Tacconelli E (2022). Determinants of persistence of symptoms and impact on physical and mental wellbeing in Long COVID: a prospective cohort study. J Infect.

[60] (2022). Coronadashboard - varianten van het coronavirus. Rijksoverheid.

[61] Nulty DD (2008). The adequacy of response rates to online and paper surveys: what can be done?. Assess Eval High Educ.

