# Supplementary material for: Evaluation of Primary Allied Health Care in Patients Recovering From COVID-19 at 6-Month Follow-up: Dutch Nationwide Prospective Cohort Study
Source: JMIR Public Health Surveill. 2023 Oct 20;9:e44155. doi: 10.2196/44155 (PMC10592721; doi:10.2196/44155)
Supplement: Multimedia Appendix 1 [file publichealth_v9i1e44155_app1.docx]

**Supplemental File 1**. Missingness at random on general outcome measures at baseline, three and six months in patients recovering from COVID-19 receiving primary allied healthcare in a Dutch prospective cohort study.

|  | Total group | Lost-to-follow-up after T0 |
| --- | --- | --- |
| Participation, *mean ± SD*  USER-P frequencies scale  USER-P restrictions scale  USER-P satisfaction scale | ^a^n = 1271  27.5 ± 10.3  65.8 ± 20.1  48.6 ± 17.8 | ^a^n = 248  27.6 ± 10.8  64.8 ± 20.8  49.3 ± 18.6 |
| Health-related quality of life  EQ-VAS, *mean ± SD* | ^a^n = 1289  55.5 ± 17.8 | ^a^n = 247  58.8 ± 17.4 |
| Fatigue  FSS mean score, *mean ± SD*  ≥4 points, *n (%)* | ^a^n = 1281  5.6 ± 1.0  1205 (94) | ^a^n = 243  5.4 ± 1.1  226 (93) |
| Physical functioning  PROMIS-PF T-score, *mean ± SD*  100% impaired, limited or restricted  80-99% impaired, limited or restricted  60-79% impaired, limited or restricted  40-59% impaired, limited or restricted  20-39% impaired, limited or restricted  1-19% impaired, limited or restricted  0% impaired, limited or restricted | ^a^n = 1279  37.7 ± 6.0  2 (0)  427 (33)  452 (35)  259 (20)  97 (8)  39 (3)  4 (0) | ^a^n = 245  38.0 ± 6.3  1 (0)  73 (30)  84 (34)  61 (25)  16 (7)  9 (4)  1 (0) |
| Psychological well-being  HADS anxiety score, *mean ± SD*  ≤7 points, *n (%)*  8-10 points  ≥11 points  HADS depression score, *mean ± SD*  ≤7 points, *n (%)*  8-10 points  ≥11 points | ^a^n = 1271  7.1 ± 4.5  746 (59)  233 (18)  292 (23)  7.3 ± 4.2  689 (54)  297 (24)  285 (22) | ^a^n = 370  7.1 ± 4.5  213 (58)  68 (18)  89 (24)  7.4 ± 4.2  202 (55)  89 (24)  79 (21) |

*Abbreviations:* USER-P: Utrecht Scale for Evaluation of Rehabilitation Participation. EQ-VAS: EuroQol Visual Analogue Scale. FSS: Fatigue Severity Scale. PROMIS: Patient-Reported Outcomes Measurement Information System. HADS: Hospital Anxiety and Depression Scale. SD: Standard Deviation.

^a^Data were not fully available for all patients: the n within the table depicts the number of patients with available data.

**Supplemental File 2**. Multivariable regression models on the outcome measures including age, sex and hospital admission status in patients recovering from COVID-19 receiving primary allied healthcare in a Dutch prospective cohort study.

| Outcome measure / factor | β | 95% CI | *P* value |
| --- | --- | --- | --- |
| Participation (USER-P frequencies scale) | **R^2^ overall model**: .277 (*P* <.001) | | |
| Age | –.057 | .045 to –.112 | .045 |
| Sex |  |  | .13 |
| *Male* | ref |  |  |
| *Female* | –1.043 | –2.392 to .306 | .13 |
| Hospital admission |  |  | .003 |
| *No* | ref |  |  |
| *Hospital ward* | 2.704 | .898 to 4.511 | .003 |
| *ICU* | 2.973 | .457 to 5.489 | .021 |
| Baseline score | –.506 | –.568 to –.443 | <.001 |
| Participation (USER-P restrictions scale) | **R^2^ overall model**: .277 (*P* <.001) | | |
| Age | –.031 | –.133 to .071 | .56 |
| Sex |  |  | <.001 |
| *Male* | ref |  |  |
| *Female* | –5.496 | –8.028 to –2.963 | <.001 |
| Hospital admission |  |  | <.001 |
| *No* | ref |  |  |
| *Hospital ward* | 3.819 | .459 to 7.179 | .026 |
| *ICU* | 0.319 | 4.646 to 13.992 | <.001 |
| Baseline score | –.460 | –.518 to –.403 | <.001 |
| Participation (USER-P satisfaction scale) | **R^2^ overall model**: .162 (*P* <.001) | | |
| Age | –.024 | –.129 to .081 | .65 |
| Sex |  |  | .003 |
| *Male* | ref |  |  |
| *Female* | –3.843 | –6.412 to –1.275 | .003 |
| Hospital admission |  |  | .05 |
| *No* | ref |  |  |
| *Hospital ward* | 2.441 | –.965 to 5.846 | .16 |
| *ICU* | 5.374 | .690 to 10.057 | .025 |
| Baseline score | –.415 | –.482 to –.348 | <.001 |
| Health-related quality of life (EQ-VAS) | **R^2^ overall model**: .070 (*P* <.001) | | |
| Age | –.003 | –.009 to .004 | .44 |
| Sex |  |  | .004 |
| *Male* | ref |  |  |
| *Female* | .241 | .075 to .408 | .004 |
| Hospital admission |  |  | .21 |
| *No* | ref |  |  |
| *Hospital ward* | –.189 | –.409 to .032 | .09 |
| *ICU* | .037 | –.269 to .344 | .81 |
| Baseline score | –.306 | –.387 to –.226 | <.001 |
| Fatigue (FSS mean score) | **R^2^ overall model**: .245 (*P* <.001) | | |
| Age | .016 | –.088 to .121 | .76 |
| Sex |  |  | <.001 |
| *Male* | ref |  |  |
| *Female* | –4.767 | –7.355 to –2.179 | <.001 |
| Hospital admission |  |  | .13 |
| *No* | ref |  |  |
| *Hospital ward* | 3.473 | .019 to 6.927 | .049 |
| *ICU* | 2.046 | –2.693 to 6.786 | .40 |
| Baseline score | –.525 | –.591 to –.460 | <.001 |
| Physical functioning (PROMIS-PF T-score) | **R^2^ overall model**: .064 (*P* <.001) | | |
| Age | –.045 | –.085 to –.005 | .001 |
| Sex |  |  | <.001 |
| *Male* | ref |  |  |
| *Female* | –2.611 | –3.636 to –1.586 | <.001 |
| Hospital admission |  |  | .002 |
| *No* | ref |  |  |
| *Hospital ward* | 1.482 | .138 to 2.827 | .031 |
| *ICU* | 3.068 | 1.214 to 4.921 | .001 |
| Baseline score | –.131 | –.209 to –.052 | .001 |
| Psychological well-being (HADS anxiety) | **R^2^ overall model**: .161 (*P* <.001) | | |
| Age | –.025 | –.048 to –.003 | .029 |
| Sex |  |  | .27 |
| *Male* | ref |  |  |
| *Female* | .315 | –.243 to .873 | .27 |
| Hospital admission |  |  | .10 |
| *No* | ref |  |  |
| *Hospital ward* | .012 | –.735 to .759 | .96 |
| *ICU* | 1.088 | .071 to 2.106 | .036 |
| Baseline score | –.356 | –.411 to –.300 | <.001 |
| Psychological well-being (HADS depression) | **R^2^ overall model**: .182 *(*P <.001) | | |
| Age | .006 | –.016 to .028 | .61 |
| Sex |  |  | .08 |
| *Male* | ref |  |  |
| *Female* | .475 | –.064 to 1.013 | .08 |
| Hospital admission |  |  | .22 |
| *No* | ref |  |  |
| *Hospital ward* | .109 | –.612 to .831 | .77 |
| *ICU* | .881 | –.105 to 1.866 | .08 |
| Baseline score | –.384 | –.443 to –.326 | <.001 |

*Abbreviations:* USER-P: Utrecht Scale for Evaluation of Rehabilitation Participation. EQ-VAS: EuroQol Visual Analogue Scale. FSS: Fatigue Severity Scale. PROMIS: Patient-Reported Outcomes Measurement Information System. SE: Standard error. CI: Confidence interval. Ref: Reference value.

**Supplemental File 3.** Clinically relevant improvement at six months follow-up of patients recovering from COVID-19 receiving primary allied healthcare in a Dutch prospective cohort study.

| Outcome measures | Baseline vs. six months follow-up, *n (%)* |
| --- | --- |
| USER-P restrictions scale  Clinically relevant improvement  No clinically relevant improvement  Clinically relevant deterioration | ^a^n = 890  576 (65)  193 (22)  121 (13) |
| USER-P satisfaction scale  Clinically relevant improvement  No clinically relevant improvement  Clinically relevant deterioration | ^a^n = 891  543 (61)  182 (20)  166 (19) |
| EQ-VAS  Clinically relevant improvement  No clinically relevant improvement  Clinically relevant deterioration | ^a^n = 908  540 (60)  255 (28)  113 (12) |
| FSS mean score  Clinically relevant improvement  No clinically relevant improvement  Clinically relevant deterioration | ^a^n = 904  490 (54)  319 (35)  95 (11) |
| PROMIS-PF T-score  Clinically relevant improvement  No clinically relevant improvement  Clinically relevant deterioration | ^a^n = 902  517 (57)  357 (40)  28 (3) |
| HADS anxiety score  Clinically relevant improvement  No clinically relevant improvement  Clinically relevant deterioration | ^a^n = 901  345 (38)  326 (36)  230 (26) |
| HADS depression score  Clinically relevant improvement  No clinically relevant improvement  Clinically relevant deterioration | ^a^n = 901  428 (47)  303 (34)  170 (19) |

*Abbreviations:* USER-P: Utrecht Scale for Evaluation of Rehabilitation Participation. EQ-VAS: EuroQol Visual Analogue Scale. FSS: Fatigue Severity Scale. PROMIS: Patient-Reported Outcomes Measurement Information System. HADS: Hospital Anxiety and Depression Scale.

^a^Data were not fully available for all patients: the n within the table depicts the number of patients with available data.

**Supplemental File 4**. Univariable regression models on outcome measures participation, health-related quality of life, fatigue, physical functioning and psychological well-being in patients recovering from COVID-19 receiving primary allied healthcare in a Dutch prospective cohort study.

1. **USER-P**
   1. Frequencies scale

| Factor | β | 95% CI | *P* value |
| --- | --- | --- | --- |
| Age  Sex  Male  Female  Hospital admission  No  Hospital ward  ICU  BMI  Normal/underweight  Overweight  Obese  Comorbidities  0  1  ≥2  Smoking status  Never  Former  Current  Baseline score | .086  ref  –2.582  ref  4.453  7.034  ref  .612  .382  ref  –.417  .303  ref  –.092  –.957  –.514 | .027 to .144  –4.021 to –1.142  2.522 to 6.384  4.270 to 9.798  –1.290 to 2.515  –1.601 to 2.365  –2.013 to 1.070  –2.004 to 2.610  –2.299 to 2.115  –4.402 to 2.487  –.572 to –.455 | .004  <.001  <.001  <.001  <.001  <.001  .82  .53  .71  .77  .55  .80  .86  .94  .59  <.001 |

- 1. Restrictions scale

| Factor | β | 95% CI | *P* value |
| --- | --- | --- | --- |
| Age  Sex  Male  Female  Hospital admission  No  Hospital ward  ICU  BMI  Normal/underweight  Overweight  Obese  Comorbidities  0  1  ≥2  Smoking status  Never  Former  Current  Baseline score | –.008  ref  –4.403  ref  5.442  15.730  ref  1.783  1.974  ref  –1.798  –1.952  ref  1.628  –.829  –.454 | –.118 to .102  –7.099 to –1.707  1.851 to 9.033  10.589 to 20.870  –1.528 to 5.473  –1.674 to 5.622  –4.676 to 1.080  –6.258 to 2.354  –2.481 to 5.736  –7.242 to 5.584  –.509 to –.399 | .89  .001  .001  <.001  .003  <.001  .46  .27  .29  .39  .22  .37  .70  .44  .80  <.001 |

- 1. Satisfaction scale

| Factor | β | 95% CI | *P* value |
| --- | --- | --- | --- |
| Age  Sex  Male  Female  Hospital admission  No  Hospital ward  ICU  BMI  Normal/underweight  Overweight  Obese  Comorbidities  0  1  ≥2  Smoking status  Never  Former  Current  Baseline score | .097  ref  –1.547  ref  1.120  5.099  ref  –.289  –.121  ref  –1.756  –.608  ref  2.185  2.306  –.387 | –.201 to .006  –4.101 to 1.007  –2.354 to 4.594  .126 to 10.072  –3.688 to 3.111  –3.664 to 3.421  –4.470 to .958  –4.669 to 3.453  –1.714 to 6.083  –3.779 to 8.391  –.449 to –.326 | .07  .24  .24  .12  .53  .045  .99  .87  .95  .45  .20  .77  .44  .27  .46  <.001 |

1. **EQ-VAS**

| Factor | β | 95% CI | *P* value |
| --- | --- | --- | --- |
| Age  Sex  Male  Female  Hospital admission  No  Hospital ward  ICU  BMI  Normal/underweight  Overweight  Obese  Comorbidities  0  1  ≥2  Smoking status  Never  Former  Current  Baseline score | –.014  ref  –4.051  ref  3.944  2.300  ref  –.556  .592  ref  –.132  –2.624  ref  3.101  –3.163  –.502 | –.123 to .096  –6.756 to –1.346  .244 to 7.644  –2.974 to 7.575  –4.092 to 2.980  –3.091 to 4.274  –3.017 to 2.752  –6.921 to 1.674  –.993 to 7.196  –9.535 to 3.264  –.563 to –.440 | .81  .003  .003  .09  .037  .39  .81  .76  .75  .48  .93  .23  .19  .14  .34  <.001 |

1. **FSS mean score**

| Factor | β | 95% CI | *P* value |
| --- | --- | --- | --- |
| Age  Sex  Male  Female  Hospital admission  No  Hospital ward  ICU  BMI  Normal/underweight  Overweight  Obese  Comorbidities  0  1  ≥2  Smoking status  Never  Former  Current  Baseline score | –.004  ref  .198  ref  –.224  .131  ref  –.035  –.106  ref  –.018  .086  ref  .011  –.234  –.313 | –.010 to .003  .040 to .355  –.437 to –.011  –.174 to .435  –.244 to .175  –.324 to .112  –.186 to .149  –.163 to .335  –.225 to .248  –.609 to .142  –.390 to –.237 | .27  .014  .014  .07  .039  .40  .62  .75  .34  .74  .83  .50  .47  .93  .22  <.001 |

1. **PROMIS-PF T-score**

| Factor | β | 95% CI | *P* value |
| --- | --- | --- | --- |
| Age  Sex  Male  Female  Hospital admission  No  Hospital ward  ICU  BMI  Normal/underweight  Overweight  Obese  Comorbidities  0  1  ≥2  Smoking status  Never  Former  Current  Baseline score | .007  ref  –2.612  ref  2.129  4.242  ref  .496  .449  ref  –.119  –.551  ref  1.039  –.074  –.108 | –.031 to .045  –3.542 to –1.682  .856 to 3.401  2.435 to 6.050  –.771 to 1.764  –.872 to 1.770  –1.125 to .886  –2.053 to .951  –.400 to 2.479  –2.322 to 2.174  –.181 to –.034 | .73  <.001  <.001  <.001  .001  <.001  .71  .44  .51  .77  .82  .47  .36  .16  .95  .004 |

1. **HADS**
   1. Anxiety score

| Factor | β | 95% CI | *P* value |
| --- | --- | --- | --- |
| Age  Sex  Male  Female  Hospital admission  No  Hospital ward  ICU  BMI  Normal/underweight  Overweight  Obese  Comorbidities  0  1  ≥2  Smoking status  Never  Former  Current  Baseline score | –.009  ref  .074  ref  .173  1.167  ref  .007  –.172  ref  –.208  –.172  ref  .104  –1.584  –.354 | –.032 to .014  –.486 to .634  –.590 to .936  .089 to 2.245  –.710 to .724  –.917 to .573  –.804 to .389  –1.047 to .703  –.732 to .941  –2.951 to –.216  –.407 to –.301 | .43  .80  .80  .10  .66  .034  .86  .99  .65  .77  .50  .70  .07  .81  .023  <.001 |

- 1. Depression score

| Factor | β | 95% CI | *P* value |
| --- | --- | --- | --- |
| Age  Sex  Male  Female  Hospital admission  No  Hospital ward  ICU  BMI  Normal/underweight  Overweight  Obese  Comorbidities  0  1  ≥2  Smoking status  Never  Former  Current  Baseline score | .014  ref  .219  ref  .408  1.335  ref  .176  .268  ref  –.166  .432  ref  –.072  –1.516  –.392 | –.008 to .036  –.327 to .765  –.335 to 1.150  .285 to 2.385  –.552 to .904  –.488 to 1.024  –.748 to .416  –.421 to 1.285  –.888 to .754  –2.851 to –.181  –.447 to –.337 | .22  .43  .43  .033  .28  .013  .78  .64  .49  .43  .58  .32  .08  .86  .026  <.001 |

**Supplemental File 5**. Multivariable regression models on outcome measures.

1. **USER-P**
   1. Frequencies scale

| Factor | β | 95% CI | *P* value |
| --- | --- | --- | --- |
| Age  Sex  Male  Female  Hospital admission  No  Hospital ward  ICU  Baseline score | –.057  ref  –1.043  ref  2.704  2.973  –.506 | –.112 to –.001  –2.392 to .306  .898 to 4.511  .457 to 5.489  –.568 to –.443 | .045  .13  .13  .003  .003  .021  <.001 |

R^2^ overall model: 0.277 (*P*<.001)

| Factor | β | 95% CI | *P* value |
| --- | --- | --- | --- |
| Age  Hospital admission  No  Hospital ward  ICU  Baseline score | –.045  ref  2.951  3.285  –.506 | –.099 to .009  1.182 to 4.720  .807 to 5.763  –.569 to –.444 | .10  .001  .001  .009  <.001 |

R^2^ overall model: 0.275 (*P*<.001)

| Factor | β | 95% CI | p-value |
| --- | --- | --- | --- |
| Hospital admission  No  Hospital ward  ICU  Baseline score | ref  2.556  3.079  –.496 | .851 to 4.262  .611 to 5.547  –.558 to –.435 | .001  .003  .015  <.001 |

R^2^ overall model: 0.272 (*P*<.001)

- 1. Restrictions scale

| Factor | β | 95% CI | *P* value |
| --- | --- | --- | --- |
| Sex  Male  Female  Hospital admission  No  Hospital ward  ICU  Baseline score | ref  –5.337  ref  3.581  9.165  –.462 | –7.813 to –2.861  .316 to 6.845  4.522 to 13.809  –.520 to –.405 | <.001  <.001  <.001  .032  <.001  <.001 |

R^2^ overall model: 0.277 (*P*<.001)

- 1. Satisfaction scale

| Factor | β | 95% CI | *P* value |
| --- | --- | --- | --- |
| Age  Hospital admission  No  Hospital ward  ICU  Baseline score | .005  ref  3.536  6.701  –.403 | –.099 to .108  .189 to 6.884  2.076 to 11.326  –.470 to –.337 | .93  .005  .038  .005  <.001 |

R^2^ overall model: 0.159 (*P*<.001)

| Factor | β | 95% CI | *P* value |
| --- | --- | --- | --- |
| Hospital admission  No  Hospital ward  ICU  Baseline score | ref  3.577  6.728  –.402 | .356 to 6.798  2.144 to 11.311  –.467 to –.338 | .003  .030  .004  <.001 |

R^2^ overall model: 0.159 (*P*<.001)

1. **EQ-VAS**

| Factor | β | 95% CI | *P* value |
| --- | --- | --- | --- |
| Sex  Male  Female  Hospital admission  No  Hospital ward  ICU  Baseline score | ref  –4.855  ref  3.594  2.106  –.524 | –7.378 to –2.333  .231 to 6.957  –2.615 to 6.827  –.589 to –.459 | <.001  <.001  .10  .036  .38  <.001 |

R^2^ overall model: 0.245 (*P*<.001)

1. **FSS mean score**

| Factor | β | 95% CI | *P* value |
| --- | --- | --- | --- |
| Sex  Male  Female  Hospital admission  No  Hospital ward  ICU  Baseline score | ref  .255  ref  –.208  .029  –.303 | .093 to .418  –.423 to .007  –.277 to .335  –.383 to –.223 | .002  .002  .15  .06  .85  <.001 |

R^2^ overall model: 0.075 (*P*<.001)

| Factor | β | 95% CI | *P* value |
| --- | --- | --- | --- |
| Sex  Male  Female  Baseline score | ref  .284  –.301 | .130 to .438  –.381 to –.222 | <.001  <.001  <.001 |

R^2^ overall model: 0.070 (*P*<.001)

1. **PROMIS-PF T-score**

| Factor | β | 95% CI | *P* value |
| --- | --- | --- | --- |
| Sex  Male  Female  Hospital admission  No  Hospital ward  ICU  Baseline score | ref  –2.342  ref  1.149  2.917  –.125 | –3.341 to –1.343  –.165 to 2.463  1.064 to 4.771  –.203 to –.046 | <.001  <.001  .004  .09  .002  .002 |

R^2^ overall model: 0.064 (*P*<.001)

1. **HADS**
   1. Anxiety score

| Factor | β | 95% CI | *P* value |
| --- | --- | --- | --- |
| Hospital admission  No  Hospital ward  ICU  Baseline score | ref  –.320  .810  –.346 | –1.026 to .387  –.184 to 1.804  –.402 to –.291 | .15  .38  .11  <.001 |

R^2^ overall model: 0.158 (*P*<.001)

| Factor | β | 95% CI | *P* value |
| --- | --- | --- | --- |
| Baseline score | –.354 | –.407 to –.301 | <.001 |

R^2^ overall model: 0.160 (*P*<.001)

- 1. Depression score

| Factor | β | 95% CI | *P* value |
| --- | --- | --- | --- |
| Hospital admission  No  Hospital ward  ICU  Baseline score | ref  –.007  .735  –.386 | –.686 to .672  –.225 to 1.695  –.444 to –.328 | .32  .98  .13  <.001 |

R^2^ overall model: 0.179 (*P*<.001)

| Factor | β | 95% CI | *P* value |
| --- | --- | --- | --- |
| Baseline score | –.392 | –.447 to –.337 | <.001 |

R^2^ overall model: 0.179 (*P*<.001)
